# Enhancing interprofessional teamwork between youth care professionals using an electronic health record; a mixed methods intervention study

**DOI:** 10.1080/13561820.2024.2314461

**Published:** 2024-02-27

**Authors:** Janine Benjamins, Emely de Vet, Annemien Haveman-Nies

**Affiliations:** a Icare JGZ, department Jeugdgezondheidszorg, Meppel, the Netherlands; b Chair group Consumption and Healthy Lifestyles, Wageningen University and Research, Wageningen, the Netherlands; c GGD Noord- en Oost-Gelderland, department Jeugdgezondheid, Warnsveld, the Netherlands

**Keywords:** Child health services, collaboration, electronic health records, interprofessional teamwork

## Abstract

We aimed to investigate whether using a shared electronic patient record (EPR-Youth) strengthened interprofessional teamwork among professionals in youth care and child healthcare. Using a mixed-methods design, we compared two partly overlapping samples of professionals, who completed questionnaires before the introduction of EPR-Youth (*n* = 117) and 24 months thereafter (*n* = 127). Five components of interprofessional teamwork (interdependence, newly created professional activities, flexibility, collective ownership of goals, and reflection on processes) were assessed for this study. Midway through the study period, focus groups were held with 12 professionals to examine how EPR-Youth contributed to interprofessional teamwork. Professionals reported significantly more flexibility after the introduction of EPR-Youth than before. Professionals scored slightly -but not significantly- more positively on the other components of teamwork. Focus group participants reported that using EPR-Youth strengthened their sense of interdependence and collective ownership of goals, and contributed to newly created professional activities. At baseline, levels of interprofessional teamwork differed between organizations. Focus group participants confirmed these differences and attributed them to differences in facilitation of interprofessional teamwork. Our findings suggest that using EPR-Youth can foster interprofessional teamwork. Organizational differences underline that implementing an EPR alone is inadequate: shared definitions of teamwork and organizational facilities are needed to strengthen interprofessional teamwork.

## Introduction

Interprofessional collaboration is considered crucial for delivering high-quality healthcare and social care. Most Western countries face increasingly complex health problems that can only be effectively addressed through collaboration between healthcare professionals, such as doctors and nurses, and non-healthcare professionals, such as social workers and occupational therapists (Bronstein, 2003; D’Amour et al., 2005). Approaching complex problems from each domain separately might cause scattered care accompanied by rising costs. This risk arises in adult health care, where comorbidities require an interprofessional approach. However, in healthcare and social care for children, interprofessional collaboration also becomes urgent. Children and families too encounter complex problems that are interconnected across different life domains, such as school, family, and community systems (Mellin et al., 2011).

Interprofessional collaboration is a heterogeneous concept, indicating an interpersonal process among healthcare workers from multiple professions (Barr et al., 2008; Bruner, 1991; Petri, 2010). *Interprofessional* collaboration involves different healthcare and social care professionals, and implies shared goals and regular interactions for negotiating and agreeing on how to solve care problems (Reeves et al., 2011). This differs from *multiprofessional* collaboration, in which professionals work alongside each other, rather than interactively. In this paper, we are focusing on interprofessional *teamwork*. The term teamwork is used when involved professionals share a team identity and work closely together in an integrated and interdependent manner. We prefer this term to *collaboration*, which does not imply a shared team identity and refers to a less integrated and interdependent working practice (Barr et al., 2008; Xyrichis et al., 2018).

**Figure 1.**
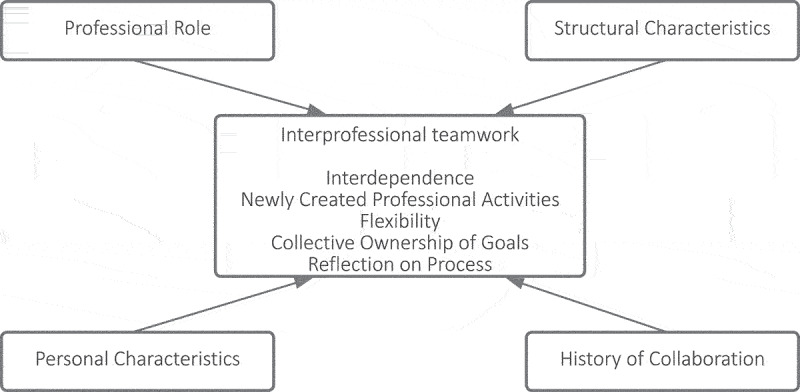
Model for interprofessional collaboration (Bronstein, 2003).

Different models have been developed to explore interprofessional teamwork. For this study, we applied the much-cited model for interprofessional collaboration by Bronstein et al., which was developed in the US context of social workers collaborating with health care professionals and draws on several theoretic frameworks (Bronstein, 2003). With an emphasis on integration and interdependence, the model describes five core components and four contributing factors of interprofessional teamwork between social workers and healthcare professionals (Iachini et al., 2018). The five core components are (Figure 1) (a) Interdependency, referring to professionals being dependent on each other to reach their goals. For example, when a child is diagnosed with attention deficit/hyperactivity disorder (ADHD), a physician prescribes medication along with parent training in behavior management (PTBM), given by a behavior therapist, and classroom interventions performed by teachers (Wolraich et al., 2019); (b) Newly created professional activities, referring to collaborative programs or structures that help professionals achieve things they could not have achieved independently. In case of ADHD-patients, multidisciplinary guidelines help professionals to keep track of each other’s role and make use of each other’s competencies (Wolraich et al., 2019); (c) Flexibility, referring to deliberate role-blurring. As opposed to strictly following the guidelines, professionals can choose to do something extra or different, when that would be helpful for this specific patient at this specific moment; (d) Collective ownership of goals, referring to a shared sense of responsibility to reach goals. A collective goal can be the health of the group of patients that professionals collaboratively care for, each one from his own perspective; (e) Reflection on process, referring to professionals thinking and talking about their collaboration to enhance the process (Bronstein, 2003). Bronstein’s model defined four factors that could influence the degree of interprofessional teamwork: (a) how professionals experience and define their “professional role”; (b) the structural characteristics of a professional’s job, such as workload, a collaboration-supportive culture, professional autonomy and how organizations facilitate collaboration with time and space; (c) personal characteristics of professionals; (d) whether professionals have a history of and positive experiences with interprofessional teamwork.

Researchers have shown that interprofessional collaboration can be strengthened by the shared use of electronic health records. Specifically, sharing information and knowledge, and drawing collaborative care plans in such an interprofessional electronic health record could enhance the quality of care (Finney Rutten et al., 2014; Lin et al., 2019; Vos et al., 2020). However, some studies point out that electronic health records can also inhibit collaboration, due to improper registration or a mismatch with working processes (Chase et al., 2014; Collins et al., 2011).

Although there is a growing body of literature on the effect of using electronic health records, there are few studies on the interprofessional use of electronic health records among adolescents, mainly because of privacy issues associated with using electronic health records in this age group (Bourgeois et al., 2018). In the Dutch North Veluwe region, youth care organizations have joined forces to tackle these privacy issues and build an interprofessional health record for youth and preventive child health care. This Electronic Patient Record, referred to as EPR-Youth, aims to integrate regional care for children and adolescents. In the Netherlands, the interprofessional use of an electronic health record between preventive child healthcare (PCH) and child social care is unique, as is a fully transparent child and adolescent health record. Although EPR-Youth provides a unique opportunity to combine registration by different professions in a client-accessible system, it is unknown whether its use will strengthen collaboration among those professions.

Therefore, our study aimed to investigate whether the use of EPR-Youth contributes to interprofessional teamwork between professionals in youth care and child healthcare. Furthermore, we aimed to contribute to the body of knowledge on the interprofessional use of electronic health records among families and adolescents.

## Methods

### Context

The Netherlands established a PCH system strongly underpinned by public health legislation (Siderius et al., 2016). Over 90% of all Dutch children follow the full free program, comprising 10 visits with a PCH physician or nurse in the first year, five visits each year between ages 1 and 4 and between 4 and 18 years. PCH professionals have a role in the prevention and early detection of physical problems, like heart conditions, growth problems, infectious diseases, and visual impairment, and social problems, such as parenting problems, behavioral or psychiatric problems, child abuse, financial issues, and substance abuse. These professionals refer to primary or secondary healthcare, mental healthcare, or to (social) youth care when needed. Approximately 10% of all children receive youth care for mental disorders, psychosocial problems, and behavioral problems (Dutch Central Bureau for Statistics, 2021). Dutch municipalities provide preventive and youth care to all children.

In the North Veluwe region, six municipalities commissioned two PCH organizations and one organization providing youth care to integrate their services in Centres for Youth and Family with interprofessional teams (Regio Noord Veluwe, 2018). Jointly, they provide preventive healthcare to 38.000 children aged 0–18 years in the region and additional youth care whenever needed. One PCH organization provided services to children aged 0–3 years (PCH 0–3), the other provided services to children aged 4–18 years (PCH 4–18). During the 3 years of this study, 58 professionals were working in PCH 0–3 and 18 in PCH 4–18. The number of youth care workers increased from 60 to 80, owing to an increasing demand for youth care. Centre for Youth and Family teams comprised a mix of child health physicians, child health nurses, youth care workers with varying backgrounds (e.g., social work, mental healthcare, or child protection services). In this paper, these professionals are referred to as “non-administrative professionals.” Each municipality had its own interprofessional and self-organizing team. These interprofessional teams met regularly to discuss complex cases or reflect on their professional behaviors. The teams were completed by administrative professionals, such as child health assistants, secretaries, screeners, and planners. The teams did not differ in composition, although the team in the municipality Harderwijk differed in size from the other teams, being twice as large because the municipality was larger than the other five. The populations of parents and children in the six municipalities did not differ significantly, in terms of ethnicity, educational level, or religion.

### Intervention

To facilitate the integration of PCH and youth care, an electronic client record was developed (EPR-Youth) that was used by all professionals working at the Centre for Youth and Family. The professionals had access only to the records of the children with whom they were involved. In the health record, they had access to all information relevant to their job, either reported by themselves or by their colleagues from other professions. When youth care professionals were involved in a case, EPR-Youth alerted the involved PCH-professionals. Parents and adolescents aged 12 years and older had access to a client portal where they could read the full content of their record. The client portal offered a view log in which parents and adolescents could see which professionals had had access to their records.

### Research design

A mixed methods research design with an explanatory sequential approach was used for this study. All professionals were invited to complete a pretest questionnaire prior to the introduction of EPR-Youth, followed by posttest questionnaires 5 and 24 months after implementation. Respondents were assigned an ID number to allow a comparison between individual pre- and posttest measurements. Due to a low response rate related to the COVID-19 pandemic (*n* = 67), the first posttest questionnaire was eventually excluded from the study. Halfway through the study, two focus groups were conducted with selected professionals. Data were collected between November 2018 and September 2021.

### Study population and inclusion

We included all administrative and non-administrative professionals working in Centres for Youth and Family (*n* = 135) and invited them to complete an online pretest questionnaire. Two years after the introduction of EPR-Youth, all professionals (*n* = 157) were invited to complete the posttest questionnaire. As a result of staff turnover, the samples only partially overlapped.

All professionals were invited to participate in the focus groups. From the professionals who expressed interest, two groups (*n* = 12) were selected through purposive sampling, ensuring that the focus groups represented all professions and organizations involved, both men and women, with different work experience levels.

### Measurements

To measure interprofessional teamwork, we translated the Index for Interdisciplinary Collaboration (IIC), a 42-item validated questionnaire by Bronstein et al (Bronstein, 2002; van ’t Hoff et al., 2020). The original questionnaire was developed and validated among social workers to investigate interprofessional collaboration among healthcare professionals. The questionnaire was based on Bronstein’s model for interdisciplinary collaboration (Figure 1) and included Bronstein’s five components of collaboration: interdependency (13 items), newly created professional activities (6 items), flexibility (5 items), collective ownership of goals (9 items) and reflection on process (9 items) (Bronstein, 2002). For the present study, the questionnaire was translated forward and backward, and phrasing was adapted to the context of Dutch care for youth [see Online supplement 1] (van ’t Hoff et al., 2020).

In the original questionnaire, all components showed good internal consistency (α=.75–.82) except for the component flexibility (α=.62). Because the Cronbach’s alpha scores in our study were similar to those in Bronstein’s study (van ’t Hoff et al., 2020), we calculated mean scores for each of the five components and one for the entire questionnaire. Mean scores were calculated when at least 2/3 of the questions for that component were completed (van ’t Hoff et al., 2020). The translated IIC used a 5-point Likert-type scale, ranging from 1 (*totally agree*) to 5 (*totally disagree*). In the original questionnaire, several items were worded negatively to reduce agreement bias among the respondents (Bronstein, 2002). To ensure that higher scores reflected a more positive attitude toward interprofessional teamwork, we reverse-coded scores for positively worded items. We also added the answer category “Not applicable.” One open-ended question about perceptions of interprofessional teamwork and questions about socio-demographic characteristics, such as sex, age, organization, profession and working experience, were added to the questionnaire.

### Focus groups

Semi-structured questions in an interview guide [Online supplement 2], based on Bronstein’s model for interdisciplinary collaboration, guided the focus groups (Bronstein, 2003). The interview guide addressed experiences with EPR-Youth in general, as well as the relationship between using EPR-Youth and interprofessional teamwork. For instance, whether and how using EPR-Youth impacts the exchange of information, harmonization of working processes, task flexibility, and shared ownership of goals.

To limit moderator bias, an experienced moderator, familiar with the organizational vision without being part of the development process of EPR-Youth guided the focus groups (Malterud, 2001; Mays & Pope, 2000). She was assisted by the main author as an observer, and a research assistant as notetaker. The duration of both focus groups was approximately 90 minutes. The groups were audio-recorded and transcribed verbatim. Subsequently, a member check was conducted with all participants to confirm transcript accuracy.

### Data analysis

Quantitative data from the online questionnaire were analyzed using IBM SPSS Statistics, version 27. Descriptive statistics were used to describe participants’ socio-demographic characteristics. Differences in respondents’ socio-demographic characteristics between the pre- and posttest measurements were tested using Pearson’s chi-square tests.

Missing data patterns were analyzed and compared between administrative and non-administrative professionals, showing that administrative professionals responded significantly more often with *Not applicable* than non-administrative professionals (8.4 vs 2.5 times in the 42-item questionnaire, *p* < .001). Furthermore, most administrative professionals reported that the questions were not relevant because they did not collaborate with their colleagues. Therefore, administrative professionals were excluded from further analyses.

Subsequently, a linear mixed model was used to analyze the difference between the pre- and posttest results for non-administrative professionals, including organization, municipality, time and the interaction between time and municipality and between time and organization as fixed factors. Respondent ID was included as random factor. Although profession, function, and work experience appeared to relate to influencing factors in Bronstein’s model (Figure 1), these variables did not contribute to the model and were excluded (Bronstein, 2003).

Qualitative data were analyzed using ATLAS.ti, versions 8 and 9. Three researchers performed a thematic analysis (Thorogood & Green, 2018), based on Bronstein’s model for interdisciplinary collaboration. Two independent researchers coded each interview transcript using a combination of inductive and deductive coding. In an iterative process between coding researchers, differences in coding were discussed, and themes were generated. Subsequently, theme interpretation was discussed with all authors, and minor modifications were made (Thorogood & Green, 2018).

### Data integration

Data were integrated using a narrative approach, *connecting* and *merging* data, and *building* on previous outcomes (Fetters et al., 2013). The data were *connected* by recruiting questionnaire respondents to participate in focus groups (Fetters et al., 2013). Data *building* occurred when questionnaire outcomes informed the focus group interview guide (Fetters et al., 2013). We *merged* data by combining and comparing the outcomes from the quantitative and qualitative analyses to reach a conclusion (Fetters et al., 2013).

### Ethical considerations and ethics approval

All methods were carried out in accordance with relevant guidelines and regulations and in compliance with the Netherlands Code of Conduct for Scientific Practice. The Social Sciences Ethics Committee of Wageningen University approved the research protocol, approval number 2018–24-Benjamins. All questionnaire respondents and focus group participants received an invitation with information about the study beforehand. Questionnaire respondents provided a written informed consent after receiving information about the study. Focus group participants provided verbal informed consent at the beginning of the focus groups. This informed consent was included in the recording and transcript of the session. All procedures were approved by our ethics committee.

## Results

**Figure 2.**
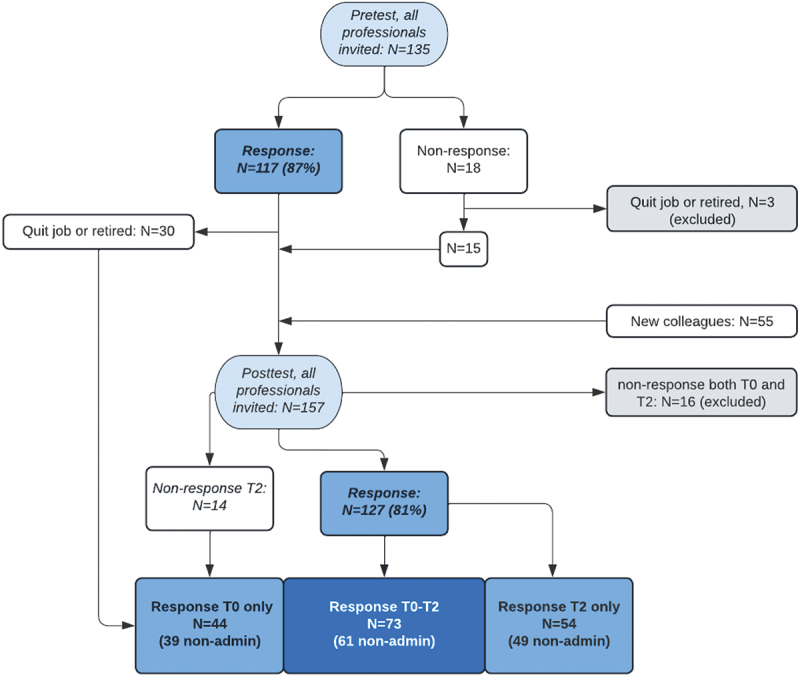
Flow diagram for inclusion of questionnaire respondents.

The response rates in the pre- and posttest were 87% (*n* = 117)and 81% (*n* = 127), respectively (Figure 2). From the pre- and posttest responses, 73 professionals completed both questionnaires. Although the professional sample changed significantly during the 3-year period, due to a high turnover rate, the respondent characteristics were mostly similar for both times (Table 1).

**Table 1.** Characteristics of respondents, in pretest and posttest, with absolute numbers and percentages.

	pre-test *n* = 117%)	post-test *n* = 127%)	*df* Pearson *χ*^2^	Two-sided *p*-value
**Sex**			0.33	.86
male	9 (7.7)	9 (7.1)		
female	108 (92.3)	118 (92.9)		
**Working experience**			2, 2.85	.24
0–10 years	41 (35·0)	57 (44.9)		
10–20 years	45 (38.5)	45 (35.4)		
>20 years	31 (26.5)	25 (19.7)		
**Profession**			7, 1.52	.98
Assistant^a^	12 (1.3)	11 (8.7)		
Behavioural expert	4 (3.4)	8 (6.3)		
PCH physcian	11 (9.4)	10 (7.9)		
Youth care Worker	58 (49.6)	62 (48.8)		
PCH Nurse	25 (21.4)	28 (22.0)		
PCH Speech therapist	2 (1.7)	2 (1.6)		
Screener^a^	3 (2.6)	3 (2.4)		
Team administrator^a^	2 (1.7)	3 (2.4)		
**Function**			2,0 .13	.94
Administrative^a^	17 (14.5)	17 (13.4)		
Youth care	62 (53.0)	70 (55.1)		
PCH^b^	38 (32.5)	40 (31.5)		
**Organisation**			2, 0.01	.97
PCH^b^ 4–18	14 (12.0)	15 (11.8)		
PCH^b^ 0–3	41 (35.0)	44 (34.6)		
Youth care	62 (53.0)	68 (53.5)		
**Municipality**			5, 1.44	.92
Oldebroek	17 (14.5)	24 (18.9)		
Elburg	18 (15.4)	18 (14.2)		
Nunspeet	20 (17.1)	24 (18.9)		
Harderwijk	37 (31.6)	39 (3.7)		
Ermelo	21 (17.9)	18 (14.2)		
Putten	4 (3.4)	4 (3.1)		

^a^Non-administrative professionals, invited for questionnaire and excluded afterward based on missing data pattern analysis.

^b^PCH = Preventive Child Healthcare.

### Self-reported interprofessional teamwork

Comparing overall mean scores, non-administrative professionals were slightly more positive about interprofessional teamwork after introduction of EPR-Youth than before, although the difference was not statistically significant (Table 2). Comparable outcomes were found for the separate components of interprofessional teamwork. Professionals were significantly more positive about flexibility after introduction of EPR-Youth than before, whereas rating of the other components improved slightly but not significantly.

**Table 2.** Total and component scores of interprofessional teamwork before and after introduction of EBPR-Youth, presented by organization, municipality, and the total research population.

	Pre-test		Post-test		Difference pre-test/posttest
	***n***	EMM* (95% *CI*)	***n***	EMM* (95% *CI*)	***F*****(*df* numerator, *df* denominator), *p*-value**
**Collaboration overall**	97	3.85 (3.74–3.95)	106	3.93 (3.82–4.04)	1.70 (1, 105.9), 0.19
Interdependence	98	4.02 (3.92–4.13)	107	4.08 (3.97–4.18)	0.60 (1, 110.2), 0.44
Newly created professional activities	92	3.70 (3.54–3.86)	101	3.80 (3.65–3.95)	0.94 (1, 126.7), 0.33
Flexibility^a^	97	3.79 (3.65–3.92)	106	4.00 (3.86–4.14)	1.97 (1, 100.7), 0.05
Collective ownership of goals	91	3.82 (3.65–3.98)	102	3.93 (3.78–4.09)	1.38 (1, 112.7), 0.24
Reflection on process	96	3.72 (3.56–3.87)	104	3.93 (3.68–3.98)	1.49 (1, 119.6), 0.23
	***n***	EMM* (95% *CI*)	***n***	EMM* (95% *CI*)	
**Organisation**					
PCH 4–18^b,c^	10	3.57 (3.31–3.82)	12	3.74 (3.50–3.98)	
PCH 0–3^b^	25	3.92 (3.76–4.09)	26	4.00 (3.84–4.16)	
youth care^c^	62	4.05 (3.94–4.15)	68	4.06 (3.96–4.16)	
**Municipality**					
Oldebroek^d,e^	14	4.14 (3.93–4.36)	22	4.06 (3.86–4.25)	
Elburg	15	3.91 (3.69–4.12)	15	3.95(3.72–4.17)	
Nunspeet^d^	16	3.69 (3.48–3.90)	20	3.81 (3.62–4.01)	
Harderwijk^e^	30	3.69 (3.53–3.85)	34	3.80 (3.65–3.94)	
Ermelo/Putten	22	3.80 (3.61–3.99)	15	4.00 (3.82–4.24)	

*Estimated marginal means (*EMM*) were calculated in a mixed model analysis, including organization, municipality, time, interaction between time and municipality, and interaction between time and organization as fixed factors, and ID as random factor.

^a^significant difference between pretest and posttest measurement, 2-sided *p*-value = 0.05, as tested with Bonferroni post-hoc.

^b–e^significant difference between these subgroups, 2-sided *p*-value <0.05, as tested with Bonferroni post-hoc.

Significant differences in self-reported collaboration were found between organizations, and between municipalities, before the introduction of EPR-Youth. Professionals working in PCH 4–18 were significantly less positive about interprofessional teamwork than youth care workers. And professionals from the municipality Oldebroek showed a more positive attitude toward interprofessional teamwork than professionals from the municipalities Nunspeet and Harderwijk. No significant differences in the overall mean score and separate components between the pre-and posttest were found for any single organization or municipality.

### Focus groups and qualitative questionnaire outcomes

In analyzing the qualitative data, we found that using EPR-Youth contributed to three of the five components of Bronstein’s model. Interdependence, collective ownership of goals and newly created activities were affected by using EPR-Youth, whereas flexibility and reflection on process were not affected by using EPR-Youth. Additionally, the contributing factor structural characteristics emerged as a relevant theme.

#### Interdependence

Professionals reported that the use of EPR-Youth strengthened their sense of interdependence, mostly in a practical way. As the system facilitated the sharing of necessary knowledge and information, professionals believed that they had become more aware of each other’s expertise and knowledge. Consequently, they found themselves better able to complement each other during the care process. Having direct access to relevant information about “who is doing what in this case” contributed to efficiency, and so did the possibility to transfer information between professions without contacting each other. Some professionals, however, did not use all the information in EPR-Youth because they did not feel free to read information that was added by other professions.

It is important that colleagues in the Centre for Youth and Family know each other and each other’s work and professional roles, to make use of each other’s expertise, involve each other when needed, and contribute to each other’s strength. (youth care Worker, Pre-test questionnaire)

I do not need to transfer the record to my colleague who provides PCH 4-18 when a child is 4 years old. And when I need information about an older child, I do not need to email to PCH 4-18 to ask them, because I can find it myself with permission from the parents. (PCH Nurse, Focus group interview 1)

#### Newly created professional activities

Developing and implementing EPR-Youth together with all professions working in the Centres for Youth and Family was considered an impactful newly created professional activity. The focus group participants reported that developing and using EPR-Youth helped them realize how working processes and procedures differed among the three organizations. Consequently, they felt an urgency to develop more “newly created professional activities,” such as synchronizing working processes and registration habits, and clarifying and describing each other’s roles and tasks.

So now we will be starting an action learning cycle in most teams, to learn with and from each other how to use EPR-Youth. We must meet each other more regularly to improve the use of this new tool.(Behavioural scientist, Focus group interview 2)

For example, sometimes my colleagues report in EPR-Youth what questions parents ask and sometimes they don’t. We have no clear working agreements whether to report this or not. I would very much like to have a guideline in which we clearly state: this is how we agree to work together. (PCH physician, Focus group interview 2)

#### Collective ownership of goals

Because EPR-Youth provided the opportunity to create a shared care plan for a child or family, in which different professions could be involved, professionals believed that EPR-Youth contributed to the collective ownership of these care plans. Every professional could register their own actions and add them to the same plan. This was in line with the regional aim to create “one plan for each family” instead of separate plans from each professional’s perspective. Moreover, professionals considered the transparency of a fully client-accessible health record to support the working relationship between clients and professionals, creating more equality and rendering more responsibility to clients then before. Consequently, ownership of care plans and goals was shared not only between different professionals, but also between clients and professionals.

I think it (EPR-Youth) can be a very powerful instrument to collaborate with parents and leave the responsibility where it belongs (youth care worker, Focus group interview 1)

This is a valuable tool to keep everyone informed. It helps prevent that, in a discussion, arrangements have been made, and you meet someone in another discussion who does not know anything about it. It helps to collaborate on the same goals and in the same direction. (PCH Nurse, focus group interview 2)

#### Flexibility

Changes in flexibility, as described by the focus group participants were related to organizational facilities rather than to using EPR-Youth. Youth care professionals reported a lack of flexibility among PCH colleagues when planning inter-professional meetings. Attending these meetings was not mandatory and was not equally facilitated by the organizations involved.

PCH 4-18 colleagues have fixed working days and schedules that are not flexible. This means that we, as youth care workers, must be even more flexible and come to the office on days off. (Youth care worker, Post-test questionnaire)

Collaboration remains difficult because different organisations do not have the same working processes or facilities. Strict schedules for PCH colleagues on the one hand, and loose methods and time investment for youth workers on the other. (PCH-worker, Post-test questionnaire)

#### Reflection on process

Using EPR-Youth did not initiate any reflection on using EPR-Youth itself or integrating the use of EPR-Youth in collaborative working processes. However, the need for reflection became clear during the focus groups, when participants concluded that they lacked knowledge about the match between the system, vision of care for youth, and actual working processes. They unanimously felt that this lack of knowledge limited EPR-Youths’ potential to strengthen interprofessional teamwork.

This confirms the need for training, not only about how the system works and what buttons you need to press, but also how we use it. How do we synchronise our working processes and how do we report correctly.(Behavioural Scientist, Focus group interview 2)

In our daily work we are limited by the fact that we don’t know all buttons and how to use them. Apparently, there are functionalities that we do not make proper use of, and you cannot benefit maximally as a team. (PCH-physician, Focus group interview 2)

#### Structural characteristics

Barriers reported by professionals were mainly associated with the factor “structural characteristics” in Bronstein’s model. Overall, professionals mentioned lack of time as an important barrier to interprofessional teamwork. Especially in the questionnaires, professionals reported (*n* = 14) that their collaboration with PCH 4–18 professionals was limited, because these colleagues were facilitated less by their organization to collaborate interprofessionally than PCH 0–3 professionals and youth care workers. Professionals believed that the PCH 4–18 organization did not provide their workers with enough time, or flexibility in their working schedules, to join meetings with colleagues from other professions. Moreover, PCH 4–18 professionals were mainly working at schools and not at the office, which was reported as another barrier to interprofessional teamwork due to lack of meeting opportunities. Finally, “being part of multiple teams” was reported to be a barrier to collaboration because professionals had to divide their attention between different teams and different interacting systems.

Working in the CJG as professionals from different organisations is problematic. There is always a risk of judging the other organisation, like: How strange that you do not get/take time for that. (Unknown, Pre-test questionnaire)

Working in the same building helps people to find each other faster and know each other’s qualities. I find it difficult that parent companies behind the three organisations shape their working processes in a different way than the Centre for Youth and Family does. (PCH-worker, Post-test questionnaire)

Perhaps my answers are not very positive, but this is because I work in different locations which limits the possibility of intensive collaboration. (PCH-nurse, post-test questionnaire)

## Discussion

In this study, we investigated whether the use of EPR-Youth contributed to interprofessional teamwork between professionals in youth care and child healthcare. Although a significant effect of using EPR-Youth on interprofessional teamwork was found only for the flexibility component, an overall slightly positive trend was confirmed by the focus group outcomes. These indicated that using EPR-Youth contributed to professionals’ sense of interdependence, collective ownership of care plans, and newly created professional activities. Additionally, the qualitative data confirmed the differences between organizations and municipalities that were found in the questionnaires and expanded on the reasons for the differences between organizations, which could, for instance, be found in their facilitation of interprofessional teamwork.

Except for the component “flexibility,” no component was rated significantly higher after the introduction of EPR-Youth, although all components showed a slightly positive trend. Contrastingly, Fukkink and van Verseveld (2020) investigated growth in interprofessional collaboration between childhood care, primary education, and youth care, analyzing four components of Bronstein’s IIC, and found a significant increase in the components “interdependence,” “reflection on process,” and “newly created professional activities” (Fukkink & van Verseveld, 2020). Another Dutch study used the Index of Interprofessional Team Collaboration for Expanded School Mental Health, an adaptation of the IIC for use in schools (Mellin et al., 2010), to assess changes in interprofessional collaboration between primary and secondary school teachers and youth care workers. They found a significant increase in the components “interdependence” and “flexibility” (Haasen et al., 2022). A possible reason for the stronger effects in these two studies might be that they focused fully on interprofessional collaboration as a new intervention, whereas in our study the involved professionals had been collaborating for 4 years (Regio Noord Veluwe, 2018), and we were assessing what introducing EPR-Youth added to existing interprofessional collaboration. Another reason could be that in these examples, the type of collaboration is different, not between healthcare and social care, but between education and social care. The collaboration between healthcare and social work professionals is not unique (Ambrose-Miller & Ashcroft, 2016). However, bringing together professionals from three organizations with different backgrounds added complexity to the situation in at least two ways. First, previous researchers have reported that interprofessional practice can be hampered by power inequality between academic and non-academic staff or between the physicians and other healthcare professionals (Bångsbo et al., 2022; Cohen; Konrad et al., 2019; Sy et al., 2023). Second, professional roles and scopes differ greatly between the healthcare and social fields, calling for more discussion on who professionals are and what they can expect from each other (Ambrose-Miller & Ashcroft, 2016).

The effect of using EPR-Youth on interprofessional collaboration could have been attenuated by the COVID-19 pandemic and the differences between the organizations involved. First, the COVID pandemic started shortly after the introduction of EPR-Youth making face to face team meeting opportunities minimal. Moreover, PCH 4–18 professionals had to prioritize working in COVID-teams (performing tests, tracking contacts, and vaccinating) over their regular work. Research shows that meeting virtually as a team impedes the development of shared mental models, conceptual frameworks, and personal relationships (Cundill et al., 2019; Morrison-Smith & Ruiz, 2020; Olson & Olson, 2000). This explains why the professionals in our study progressed slowly with the creation of new professional activities, such as the synchronization of working processes and registration habits, although they felt this was urgent. Most likely, the COVID-19 pandemic impeded the process of interprofessional collaboration in general, and the effect of ERP-Youth on interprofessional collaboration more specifically. Therefore, we expect that EPR-Youths contribution to interprofessional collaboration will increase now, since the impact of the COVID-19 pandemic on working conditions decreases.

Second, the significant differences in attitudes toward interprofessional behavior among professionals from different organizations suggest that these organizations play a role in supporting interprofessional collaboration. Bronstein’s model confirms this, describing “structural characteristics” as facilitating factors provided by organizations that contribute to interprofessional collaboration (Bronstein, 2003). These include a collaboration-supportive agency culture, space for professional autonomy, and the facilitation of collaboration with time and space. The observed differences between the attitude of professionals from different organizations might be caused by different organizational views on collaboration. The respondents said that PCH 4–18 professionals were provided with less space and time to collaborate with their colleagues in the centers for youth and family than other professionals.

In the Introduction, we described the different definitions used in interprofessional practice, and how, in multiprofessional teamwork, different professions work alongside each other, performing their jobs independently (Barr et al., 2008; Choi & Pak, 2006; Stember, 1991; Xyrichis et al., 2018). Multiprofessional teams consult about the same client but do not develop a cohesive care plan, as each team member uses their expertise to develop individual care goals. In interprofessional teamwork, on the other hand, goals can only be achieved through the interactive effort of the involved professionals (Bronstein, 2003; Thylefors et al., 2005). This type of collaboration requires a high level of communication, collective decisions, goal setting, and mutual planning. In our study, although all three organizations had committed themselves to interprofessional teamwork beforehand, one organization chose a more multiprofessional approach, presumably based on different interpretations of the term interprofessional teamwork.

A shared definition of teamwork among professions, or in this case, even organizations, is important to succeed (Etherington et al., 2021). Based on such a shared definition, a shift in organizational behavior is needed, away from traditional identities, leadership models, and decision-making roles (Ahuja, 2022; Hummell et al., 2022; Rawlinson et al., 2021). New behavior should facilitate a professional’s participation in and decision-making within interprofessional interorganizational teams (Ahuja, 2022; Rawlinson et al., 2021).

We also found differences between professionals working in different municipalities that could not be explained by differences in structural characteristics, such as workload and facilities, or differences in team composition. However, the self-organizing character of every municipal team could explain some of the differences between them. Moreover, team size could have been a limiting factor in the Harderwijk team, as it was twice as large as the other teams (Lim et al., 2014). Bronstein’s model includes more factors that influence interprofessional collaboration, such as “a strong sense of professional role,” “personal characteristics” and a “history of collaboration” (Bronstein, 2003). Other authors have described different factors at the professional or team level that contribute to interprofessional collaborations, such as leadership, transparent team roles and interactive communication (Müller et al., 2014; Mulvale et al., 2016). These factors, which we did not study, may explain the differences between professionals in different municipalities. However, further research is required to identify relevant factors.

Professionals reported that sharing knowledge and information made them more aware of each other’s competencies and enabled them to complement each other during the care process. As previous researchers have emphasized, getting to know each other’s work is necessary to enhance inter-professional practice (Bångsbo et al., 2022; Pratt et al., 2018). This could sometimes conflict with legal requirements, prohibiting information exchange between organizations and professions. In our study, some professionals refrained from reading each other’s reports, because they were unsure if they were legally allowed to do so (Auschra, 2018). However, the potential benefits of knowledge sharing require finding acceptable solutions for these privacy issues. In our study, a clear agreement was reached when professionals could read each other’s information and what they could read.

This study has several strengths and limitations. The mixed-methods design, in which qualitative data expanded on repeated quantitative measurements, was a strength, generating an overview of different perspectives on interprofessional collaboration. We were limited by the exclusion of one measurement due to COVID-19 and high staff turnover. High staff turnover also limited the number of professionals who completed both questionnaires. Using a mixed model analysis, we optimized data use and were able to retain all responding participants included in the study. Another limitation was the lack of a control group. However, there was no Dutch region with a similar context in which PCH and youth care collaborate in a similar fashion without the use of a shared health record. Ultimately, the interpretation of the term interprofessional, which apparently differed among the three organizations involved, was also a limitation. This caused differences in how professionals’ collaboration was facilitated, which likely inhibited team processes.

## Conclusions

Our results suggest that using EPR-Youth can foster interprofessional teamwork between professionals in PCH and youth care. The possible effects, however, have probably been attenuated by the impact of COVID-19 and differences between the organizations involved. These differences underline that implementing an EPR alone does not contribute to interprofessional teamwork; a shared definition of teamwork and alignment of organizational facilities are needed to strengthen interprofessional teamwork. Additional research is needed to identify the factors contributing to the differences between municipal teams. This provides initial evidence of how the interprofessional use of electronic health records contributes to interprofessional care for families and children.

## Supplementary Material

Supplemental Material

## Data Availability

The data generated during and/or analyzed during the current study are deposited in the DANS EASY repository, DOI 10.17026/dans-zxq-c63v. The following data are included: data professional questionnaire, both in Excel and SPSS, a data dictionary defining each field in the set, and the logbook. Since focus group transcripts contained sensitive information, these were not included. All included data will be available after an embargo period of one year. Until then, the datasets can be retrieved upon request from the corresponding author.
