# Supplementary material for: Enhancing interprofessional teamwork between youth care professionals using an electronic health record; a mixed methods intervention study
Source: J Interprof Care. 2024 Feb 27;38(3):553–63. doi: 10.1080/13561820.2024.2314461 (PMC11018063; doi:10.1080/13561820.2024.2314461)
Supplement: Supplemental Material [file IJIC_A_2314461_SM4558.zip › Revised version supplementary material 1.docx]

Additional file 1: questionnaire, based on Index of Interdisciplinary Collaboration.

Explanatory text: when you read ‘my CYF-colleagues’ (=my colleagues in the Centre for Youth and Family), we specifically mean the colleagues working for the two organizations within the Centre for Youth and Family that are not your own. Youth care professionals: think of all PCH-professionals, PCH-professionals: think of your Youth Care colleague and your PCH-colleague who is working in the other PCH-organization. When you read ‘my colleagues; (mijn vakgenoten), we mean the colleagues with the same profession that you have (e.g. doctor, nurse, youth care worker, screener).

Interdependency

1. I utilize my CYF-colleagues for their specific expertise.

2. I consistently give feedback to my CYF-colleagues.

3. My CYF-colleagues utilize me and my colleagues for a range of tasks.

4.* Teamwork with my CYF-colleagues is not important in my ability to help parents and adolescents.

5.* My CYF-colleagues and I rarely communicate.

6. The CYF-colleagues with whom I work have a good understanding of the distinction between my role and their role(s).

7.* My CYF-colleagues make inappropriate referrals to me.

8. I can define those areas that are distinct in my professional role from that of the CYF-colleagues with whom I work.

9. I view part of my professional role as supporting the role of others with whom I work

10. My CYF-colleagues refer to me often.

11.* Cooperative work with colleagues from other disciplines is not a part of my job description

12.* My CYF-colleagues do not treat me as an equal.

13. My CYF-colleagues believe that they could not do their jobs as well without the assistance of social workers.

Newly created professional activities

14. Distinct new programs emerge from the collective work of all colleagues within the CYF.

15. Organizational protocols within the CYF reflect the existence of cooperation between professionals from different disciplines

16. Formal procedures/mechanisms exist for facilitating dialogue between professionals from different disciplines (i.c. at structured dialogue sessions or case discussions)

17.* I am not aware of situations in my CYF (or regionwide) in which a coalition, task force or committee has developed out of interdisciplinary efforts.

18. Working with CYF-colleagues leads to outcomes that we could not achieve alone.

19. Creative outcomes emerge from my work with CYF-colleagues that I could not have predicted.

Flexibility

20. I am willing to take on tasks outside of my job description when that seems important.

21.* I am not willing to sacrifice a degree of autonomy to support cooperative problem solving.

22. I utilize formal and informal procedures for problem solving with my CYF-colleagues.

23.* The CYF-colleagues with whom I work stick rigidly to their job descriptions.

24. My CYF-colleagues and I work together in many different ways.

Collective ownership of goals

25. CYF-colleagues with whom I work encourage family members’ participation in the care process.

26.* My CYF-colleagues are not committed to working together.

27. My CYF-colleagues work through conflicts with me in efforts to resolve them.

28. When CYF-colleagues make decisions together they go through a process of examining alternatives.

29. My interaction with my CYF-colleagues occurs in a climate where there is freedom to be different and to disagree.

30. Our customers (parents/adolescents) participate in interdisciplinary planning that concerns them.

31. Colleagues from all professional disciplines take responsibility for developing care plans

32*. Colleagues from all professionals disciplines do not participate in implementing care plans

33. My CYF-colleagues and I are straightforward when sharing information with parents and adolescents.

Reflection on process

34. My CYF-colleagues and I often discuss different strategies to improve our working relationships.

35. My CYF-colleagues and I talk about ways to involve other professionals in our work together

36*. My CYF-colleagues do not attempt to create a positive climate in the CYF.

37. I am optimistic about the ability of my CYF-colleagues to work with me to resolve problems

38. I help my CYF-colleagues to address conflicts with other professionals directly.

39. My CYF-colleagues are as likely as I am to address obstacles to our successful collaboration

40. My CYF-colleagues and I talk together about our professional similarities and differences including role, competencies and stereotypes.

41*. My CYF-colleagues and I do not evaluate our work together

42. I discuss with my CYF-colleagues the degree to which each of us should be involved in a particular case.

Respondents were choosing from the following answering categories: totally agree (1), agree (2), neutral (3), disagree(4), totally disagree (5), not applicable(6)

* These questions have been worded reversely, to reduce respondent agreement bias.
