# Supplementary material for: Enhancing interprofessional teamwork between youth care professionals using an electronic health record; a mixed methods intervention study
Source: J Interprof Care. 2024 Feb 27;38(3):553–63. doi: 10.1080/13561820.2024.2314461 (PMC11018063; doi:10.1080/13561820.2024.2314461)
Supplement: Supplemental Material [file IJIC_A_2314461_SM4558.zip › Supplementary material 2.docx]

Additional file 2: semi-structured interview guide for focus groups

Interview scope:

How are professionals experiencing the impact of the use of EPR-Youth on interdisciplinary collaboration?

How do professionals feel about a client-accessible health record, what positive and negative connotations do they have?

Topic list:

In general: what experiences can you describe, using EPR-Youth?

- Positive experiences: what is working well, in what way is EPR-Youth helpful?
- Negative experiences: what could work better, what is not helping? Do you have any suggestions for improvement?

Collaboration:

- Does the use of EPR-Youth contribute to interdisciplinary collaboration? In what way?
- Impact on sense of interdependency (e.g. exchanging knowledge and information, making use of each other’s expertise)
- Impact on newly created professional activities (e.g. harmonizing working processes, new interprofessional working agreements)
- Impact on flexibility (e.g. shifting tasks from one discipline to another when needed)
- Impact on shared ownership of goals (e.g. shared care plans)
- Impact on reflection on process (e.g. interdisciplinary meetings to reflect jointly on cases or working agreements)
- Impact on communication
- Impact on efficiency

How do you use EPR-Youth?

- Read information that others wrote?
- Inform clients about interdisciplinary shared use?
- Communicate with colleagues through the system?
- Disclose relevant information for colleagues if it is only visible for you?
- What could be needed to enhance collaboration?
- What could you do?

What do you need from managers and staff?

What needs to be adapted in EPR-Youth
